# A Retrospective Data Analysis for the Risk Evaluation of the Development of Drug-Associated Jaw Necrosis through Dentoalveolar Interventions

**DOI:** 10.3390/ijerph19074339

**Published:** 2022-04-04

**Authors:** Mayte Buchbender, Charlotte Bauerschmitz, Sebastian Pirkl, Marco R. Kesting, Christian M. Schmitt

**Affiliations:** Department of Oral and Maxillofacial Surgery, Friedrich-Alexander-Universität Erlangen-Nürnberg (FAU), Glückstraße 11, 91054 Erlangen, Germany; charlotte.bauerschmitz@uk-erlangen.de (C.B.); pirkl.sebastian93@gmx.net (S.P.); marco.kesting@uk-erlangen.de (M.R.K.); schmitcn@outlook.de (C.M.S.)

**Keywords:** oral surgery, antiresorptive therapy, risk profile, MRONJ

## Abstract

This study aimed to analyse the development of medication-related osteonecrosis of the jaw (MRONJ) in patients who underwent surgical intervention to identify potential risk factors between three different groups sorted by the type of oral surgery (single tooth extraction, multiple extraction, osteotomy). Data from patients with this medical history between 2010 and 2017 were retrospectively analysed. The following parameters were collected: sex, age, medical status, surgical intervention location of dentoalveolar intervention and form of medication. A total of 115 patients fulfilled the criteria and underwent 115 dental surgical interventions (female *n* = 90, male *n* = 25). In total, 73 (63.47%) of them had metastatic underlying diseases, and 42 (36.52%) had osteoporotic ones. MRONJ occurred in 10 patients (8.70%) (female *n* = 5, male *n* = 5). The occurrence of MRONJ was significantly correlated (*p* ≤ 0.05) with the mandible site and male sex. Tooth removal at the mandible site remains the main risk factor for the development of MRONJ. The risk profile of developing MRONJ after dentoalveolar interventions could be expected as follows: tooth osteotomy > multiple extractions > single tooth extraction.

## 1. Introduction

Antiresorptive drugs are no longer indispensable in medical practice. They are used in various benign and malignant diseases of the bone as adjuvant therapy. The main indications include multiple myeloma, osseous metastasised mammary or prostate cancer and primary or secondary osteoporosis. We need to distinguish between amino and non-amino-bisphosphonates [1].

Although the mechanism is not fully understood yet, nitrogen-containing/ amino- bisphosphonates, e.g., zoledronate, are metabolised via the mevalonate pathway. In contrast, non-nitrogen-containing/non-amino bisphosphonates, e.g., clodronate, are degraded in osteoclasts to form methyl-containing analogues of adenosine triphosphates. The drugs intercalate with bone metabolism, binding calcium ions and thereby preventing osteoclasts and thus bone resorption of calcified bone [2]. Thus, both groups inhibit bone resorption by suppressing osteoclasts, and the process of bone regeneration is postponed in favour of degradation [3,4,5,6]. Since antiresorptive drugs (such as denosumab, a RANKL inhibitor), and also antiangiogenetic, anti-VEGF monoclonal antibodies (such as Bevacizumab), tyrosine kinase inhibitors (such as Sunitinib) and mTOR inhibitors (such as Sirolimus) have been on the market, some of these drugs were observed to induce necrosis of the jaw, too [6,7,8]; therefore, in 2014, AAOMS created a new definition: medication-related osteonecrosis of the jaw (MRONJ) [3,9,10]. In 2017, the Japanese Allied Committee on Osteonecrosis of the Jaw published a new position paper, an update of the 2010 published paper, which described a new definition called antiresorptive agent-related osteonecrosis of the jaw (ARONJ) [11]. In the literature, both definitions, ARONJ and MRONJ, can be found equally. In this report, however, MRONJ is used as in the following [10,11,12]. 

In 2003, Marx et al. first described necrosis of the jaw under bisphosphonate [5]. Since then, subsequent studies have shown that amino- bisphosphonates play a leading role in jaw necrosis [5]. In the current literature and guidelines, the following conditions have been formulated to clearly define bisphosphonate-related necrosis of the jaw (BRONJ): the jawbone is exposed for more than 8 weeks during or after bisphosphonate therapy, and there is no radiation to the head and neck area [3,12]. The Association of Oral and Maxillofacial Surgeons (AAOMS) divided the clinical appearance of necrosis into the following four stages: Stage 0: no clinical signs of necrotic bone, but there are radiographic signs; Stage 1: exposed bone or fistula; and Stages 2 and 3: in addition to exposed bone, there is pain, redness, pus or even pathological fractures or extraoral fistulas [10].

In addition, patients can be assigned to three different risk profiles based on the form or duration of drug application [9,13,14]. Low-risk patients with primary osteoporosis (oral administration/intravenous (IV) every 12 months) are assessed at a prevalence of 0.1%. Patients with a median risk have secondary osteoporosis (IV every 6 months) with a prevalence of 1%. Patients with bony metastasis or multiple myeloma (IV every 4 weeks) are assigned a high-risk profile with a prevalence of 1–19% [13,14].

To prevent MRONJ, some caveats should be noted during surgery, such as perioperative antibiotics until complete wound healing, primary wound closure without tension, removal of sharp bone edges and minimal denudation of the periosteum [8,15,16,17]. The positive effect of drug holidays on reducing the occurrence of MRONJ is a controversy discussed in the literature [16,17,18]. In an animal study, Otto et al. showed that a drug holiday before and after tooth extraction combined with the caveats could reduce the occurrence of MRONJ after tooth extraction [16]. Ottesen et al. have not proved that a drug holiday of high-dose antiresorptive medication prevents MRONJ after tooth extraction [19].

Various parameters such as dental infection or inflammation, reduced bone remodelling, the oral microbiome, inhibition of angiogenesis, soft tissue toxicity and changed immunity, as well as some systemic diseases such as rheumatoid arthritis or diabetes mellitus, are discussed in the literature as triggers for the occurrence of MRONJ [6,9,20,21,22,23]. Bruises due to prostheses or periodontal therapies can cause MRONJ, and new studies have shown that it is not the decayed tooth itself that causes MRONJ but the actual surgical procedure. In up to 61% of cases, tooth removal is the main cause of MRONJ [10,24]. However, it should be noted that Chang et al., among others, described a higher occurrence of MRONJ predominantly after the removal of periodontal or periapical damaged teeth compared to the removal of healthy teeth. However, periapical lesions without subsequent tooth removal seem to be crucial in the development of MRONJ [6,23]. Whether further risk factors for the development of MRONJ can be considered remains unclear according to the current data. Therefore, this retrospective study highlights the association of local and systemic risk factors for the development of MRONJ.

## 2. Materials and Methods

### 2.1. Study Design and Setup

This study reports retrospective analyses of data. The ambulatory patient collective under antiresorptive therapies in the Department of Oral and Maxillofacial Surgery, University of Erlangen-Nuremberg, Germany, between 2010 and 2017 was screened. Patients who received antiresorptive therapy and needed tooth removal were included. The exclusion criteria were radiation to the head and neck area, pregnancy and being a minor. The cohort was divided into three groups based on the dentoalveolar intervention: single tooth extraction (extraction of one tooth within one quadrant), multiple extraction (all interventions with extractions of ≥2 teeth within one quadrant) and tooth osteotomy (all interventions with rotating surgical removal of the tooth). In addition, all patients with the following case histories were included in the “risk group”: malignant underlying disease, intravenous or subcutaneous application of the antiresorptive agent, high frequency of application every 4–6 weeks and >15 months of ongoing antiresorptive therapy. The screening process for the patients was performed with the help of patient charts according to the inclusion/exclusion criteria. Ethical approval (No. 38_18Bc) was obtained from the ethics committee of the medical faculties of the Friedrich-Alexander-Universität Erlangen-Nürnberg, Germany. We aimed to analyse the possible risk factors that influence the occurrence of MRONJ. Every patient who was included had a first-line surgical intervention (no revision) in the Department of Oral and Maxillofacial Surgery. Every procedure was performed under local anaesthesia (Ultracain^®^ UDS; adrenaline 1:200.000; Sanofi-Aventis GmbH, Frankfurt, Germany) and following the precautions to avoid MRONJ. The patients received a perioperative antibiotic agent (Augmentan^®^ 875 mg/125 mg; Western Pharma GmbH, Quettingen, Germany or clindamycin 600 mg, HEXAL AG, Holzkirchen, Germany) and primary local saliva-tight wound closure after eliminating the sharp bone edges after tooth extraction [25].

### 2.2. Data Extraction (Parameters/Examination)

We analysed the patients according to the following parameters:

1. Sex and age;
2. Underlying disease treated with antiresorptive drugs;
3. Co-factors (other diseases);
4. Tooth removal (single tooth extraction, multiple extraction and tooth osteotomy, localisation, number of extracted teeth);
5. Application type, dosage, duration and frequency of the antiresorptive agent;
6. Duration and start of perioperative adjuvant antibiotics;
7. Treatment of the developed MRONJ and recurrence.

### 2.3. Outcomes

Our primary outcome was defined as the risk of developing MRONJ after different surgical interventions, meaning single tooth extraction, multiple extractions and tooth osteotomy. As secondary outcome parameters, we evaluated the underlying antiresorptive treated diseases, other diseases as potential risk factors, the application type, dosage and frequency of the antiresorptive agent, the duration and start of perioperative adjuvant antibiotics, the number of extracted teeth, the localisation of surgical intervention and the treatment of the developed MRONJ and recurrence.

### 2.4. Data and Statistical Analysis

Two-sided, adjusted *p* values ≤ 0.05 were considered to be significant. The analyses were performed using SPSS 22 for Mac OS (IBM Inc., New York, NY, USA). The association of each variable with MRONJ occurrence was analysed with the nonparametric Mann–Whitney U test for ordinal variables and with the Fisher’s exact test or chi-square tests for categorical variables. After reaching the significance level, logistic regression was performed for a more precise determination.

## 3. Results

### 3.1. Patient Cohort

A total of 115 patients with *n* = 115 surgical interventions were included (Table 1). The mean age of the treated patients was 68.09 ± 11.40 years. The median age was 70 years. The youngest patient was 41 years old, and the oldest patient was 96 years old. There was no statistically significant correlation between age and the occurrence of MRONJ (*p* = 0.157, Table 2), even though the older the patient was, the lower the probability of MRONJ occurrence was. The patient cohort consisted of 90 women and 25 men, with a significantly higher likelihood of occurrence for men to develop MRONJ after tooth extraction (*p* = 0.033, Table 2). All of the patients were of Caucasian ethnicity.

### 3.2. Primary Outcome—Occurrence of Medication-Related Osteonecrosis of the Jaw (MRONJ) after Surgical Intervention

**Figure 1:**
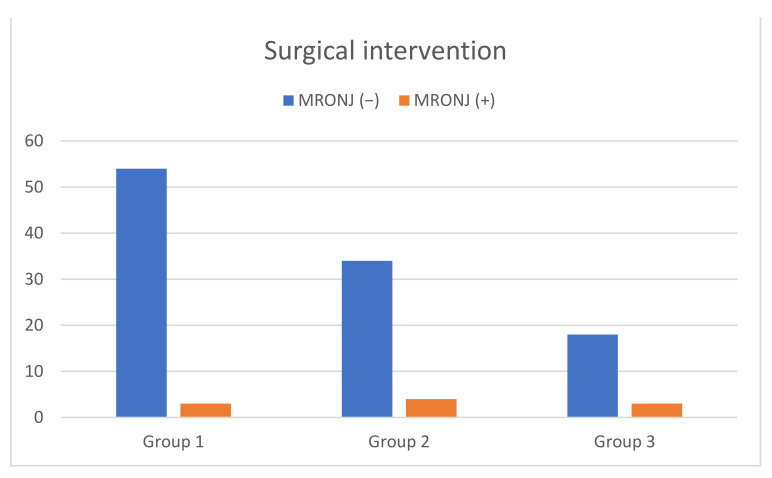
MRONJ among the three different types of surgical interventions (single tooth extraction (5.36%) (*p* = 0.323); multiple extraction 10.53% (*p* = 0.728); osteotomy 14.29% (*p* = 0.387).

**Figure 2:**
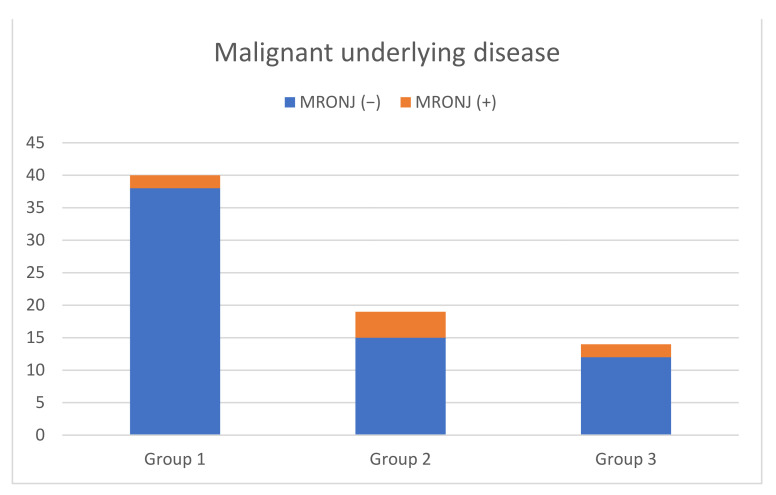
Occurrence of MRONJ among patients with malignant underlying disease depending on the surgical intervention. (*p* = 0.165).

**Figure 3:**
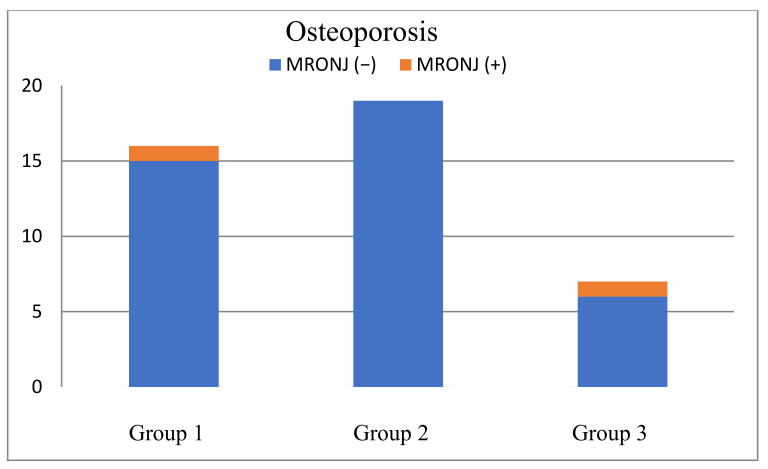
Occurrence of MRONJ among osteoporosis patients depending on surgical intervention. (*p* = 0.297).

From 115 surgical interventions, 10 cases of MRONJ (8.7%) were recorded after tooth extraction (illustrated in Table 1). Six of the patients with MRONJ were diagnosed with an exposed jawbone, and four of the patients were diagnosed with non-healed extraction sites. Moreover, there was a patient who developed MRONJ because of a pressure ulcer from the prosthesis and another patient because of a fracture, but neither was included because the aim of this study was to analyse the occurrence of MRONJ after dentoalveolar interventions. The exact percentage of single tooth extractions (Group 1), multiple extractions (Group 2), tooth osteotomies (Group 3) and the occurrence of MRONJ with its significance levels among the groups and 115 interventions are shown in Table 1 and Figure 1. Comparing Group 1 with Group 2, Group 3 with Group 1 and Group 3 with Group 2, no significant occurrence of MRONJ could be registered. As illustrated in Figure 2 and Figure 3, no significant correlations could be detected among patients with malignancies (*p* = 0.165) or osteoporosis (*p* = 0.297) who developed MRONJ after surgical intervention. The risk of developing MRONJ among those with malignant diseases was higher after multiple extractions (*n* = 4) than after single tooth extractions (*n* = 3)), but the difference was not statistically significant (*p* = 0.165, Figure 2. Looking at the surgical intervention in the osteoporosis group, there was no significant difference either (*p* = 0.297, Figure 3). Within the tooth osteotomy group, the risk was the same between malignant (*n* = 2) and osteoporotic (*n* = 1) diseases, with *p* = 1.000. The surgical interventions after the occurrence of MRONJ and patient-related details are illustrated in Table 3. In another eight patients, wound healing disorders after tooth extraction were recorded, which healed by secondary intention without the occurrence of MRONJ.

### 3.3. Secondary Outcomes

#### 3.3.1. Antiresorptive Treated Underlying Disease

In the total cohort (*n* = 115), 73 (63.47%) patients had malignancies, and 42 (36.52%) had osteoporosis (within this group, 2 had secondary osteoporosis). Of the ten recorded patients with MRONJ, eight had a malignant underlying disease (Table 3). Among them, two were patients with breast cancer, one with prostate carcinoma, three with other malignant carcinoma and two with multiple myeloma (Table 3). Two of the ten patients with MRONJ had osteoporosis, as shown in Table 3. There were statistically significant correlations between the underlying disease and the manifestation of MRONJ (*p* < 0.001, Table 2). In comparison to breast cancer, only renal cell carcinoma showed a significant correlation with MRONJ (*p* = 0.009) in the logistic regression (Table 2). Of the 115 patients, 56 (48.69%) received zoledronate, 31 (26.95%) received alendronate, 10 (8.69%) received denosumab, 9 (7.82%) received ibandronate, 7 (6.08%) received pamidronate and 2 (1.73%) received risedronate. This corresponds to the distribution of underlying diseases (Table 1 and Table 3). No statistically significant correlation was recorded for the occurrence of MRONJ under denosumab therapy (*p* = 0.077) compared to zoledronate therapy (Table 2). With the chi-square test shown in Table 2, there was no statistically significant correlation between the occurrence of MRONJ and the form of application (*p* = 0.051, Table 2). The mean duration of medication use was 46.60 months and varied from 2 months to 156 months with no statistical significance (*p*= 0.859, Table 2). The frequency of application had a statistically significant correlation with the occurrence of MRONJ (*p* = 0.038, Table 2). However, the application every four to six weeks had no statistical significance compared to a yearly application (*p* = 0.226).

#### 3.3.2. Co-factors (Other Diseases)

The comorbidities of the patients were documented, as shown in Table 1. Seven of the ten patients with MRONJ had arterial hypertension with statistical significance for MRONJ (*p* = 0.035, Table 2). There were two MRONJ patients with nicotine abuse, which was significant for the occurrence of MRONJ (*p* = 0.037). No other correlation between MRONJ and co-diseases was shown.

#### 3.3.3. Duration and Start of Perioperative Adjuvant Antibiotics

All patients received adjuvant antibiotic therapy during the surgical intervention. Only one patient denied antibiotic therapy. The mean preoperative antibiotic therapy duration was 2.92 ± 2 days. The postoperative therapy varied between four and 18 days, with no statistically significant correlations between the occurrence of MRONJ and preoperative antibiotic duration (*p* = 0.497) or postoperative antibiotic duration (*p* = 0.731, Table 2).

#### 3.3.4. Number of Extracted Teeth and Localisation of Tooth Removal

In total, 255 teeth were extracted. No statistically significant correlation was found (*p* = 0.217) between the occurrence of MRONJ and the number of extracted teeth. The extraction site was the mandible in *n* = 59, 51.30% of the 115 patients, the maxilla in *n* = 47, 40.86% of the patients, and at both sites (maxilla and mandible) in *n* = 9, 7.82% of the patients. Nine of the ten cases of MRONJ occurred in the mandible, and one case occurred in the maxilla, which corresponded to a significant occurrence of MRONJ in the mandible with *p* = 0.047 and *p* = 0.022 (Table 2). However, there were no statistically significant correlations between the anterior and posterior extraction sites and the occurrence of MRONJ (*p* = 0.849) (Table 2).

#### 3.3.5. Treatment of the Developed MRONJ and Recurrence

The surgical treatment consisted of modelling osteotomy (removing sharp edges of the bone) and primary saliva-tight wound closure in nine of the 10 patients. In three of them, complications and recurrences led to a second (*n* = 1) and a third (*n* = 2) surgical intervention, as shown in Table 3. The 10th patient diagnosed with MRONJ was only treated with oral antibiotics and local bone-edge smoothing without recurrence.

## 4. Discussion

The number of drug-associated osteonecrosis cases has increased steadily since the first cases were reported in 2003 [5,26,27]. However, an exact prevalence in the literature is lacking, and the reported prevalence rates vary between 0.3% and 6.7% depending on the dosage and form of medication administration [14,28,29]. 

Moreover, the reasons behind the development of MRONJ are often discussed in the literature. Therefore, this retrospective study was set up to identify potential risk factors. In most cases, MRONJ occurs after invasive surgical measures but can also occur spontaneously, as in a large number of cases [25,30,31]. With regard to tooth extraction as a surgical intervention, it has recently been discussed that it is not the tooth extraction itself but the apical or periodontal inflammation of the tooth that is causally valid for MRONJ [11,29,32]. This theory was explored by a recent study from 2018 that assessed periodontal status by panoramic images and showed a correlation between the risk of MRONJ and advanced periodontal loss (30% and more) [33].

Other studies showed that tooth extraction is the main risk factor for MRONJ, with a described 16-fold and up to a 33-fold increase in the risk for MRONJ [3,8,34]. In a meta-analysis, tooth extraction was indicated as a risk factor in 67% of cases [35]. Approaches such as tooth extractions, low bone turnover influenced by drugs, and previous infections or impairment of soft tissue healing are controversial causes of MRONJ [36]. 

We considered different trigger factors, such as extraction of teeth, other surgical interventions or underlying diseases, in our retrospective study. The variety in factors and the study design made it difficult to evaluate periodontal status. In our patient population, the main risk factor was the extraction of teeth. Without significant results regarding the local factors of a surgical intervention, but considering the current literature, the risk of developing MRONJ after tooth removal should be seen as follows: osteotomy (14.29%) > multiple extractions (10.53%) > single tooth extraction (5.36%). Furthermore, our data analysis showed, although without statistical significance, that the risk of MRONJ in malignancies is higher after multiple extractions than after single tooth extraction, regardless the exact number of teeth or location in the jaw (anterior vs. posterior). However, the frequency of application had a statistically significant correlation to the occurrence or MRONJ.

There are a variety of assumptions that could justify this.

On the one hand, we know from the literature that due to malignancies, the medication is usually administered intravenously or subcutaneously and the frequency is also higher. In particular, patients who received nitrogen-containing bisphosphonates intravenously due to a malignant underlying disease show an incidence rate of 18% for MRONJ [33,37,38,39,40,41]. In contrast, the incidence of MRONJ in patients with osteoporosis who received oral drug administration is much lower, between 0–0.4% [10]. We know that intravenous bisphosphonates accumulate 142.8 times faster than oral bisphosphonates, which explains the high incidence of MRONJ [42]. 

On the other hand, the wound surface increases with the number of teeth removed and the risk of wound dehiscence or wound healing disorders increases as a result. Moreover, osteonecrosis after tooth extraction occurred significantly more often in the lower jaw than in the upper jaw, which is in line with the figures in the literature [3,4,43]. Whereas in the past, it was assumed that the upper jaw was more susceptible to necrosis due to its anatomy, we now know that due to the increased bone remodelling in the lower jaw (2-fold higher than in the upper jaw), necrosis is suppressed more strongly by antiresorptive medication in the lower jaw and favours MRONJ [10,24,31,43]. Moreover, a significant correlation in the retrospective analysis was detected in favour of the male sex, which is not according to current data [44]. However, due to the study design and unequal group distribution, no evidence can be derived. 

In our study, no drug holiday was carried out due to the controversial discussion in the literature about the success of interrupting antiresorptive (AR) therapy. In addition, the exact and meaningful timing of a drug holiday has not been fully clarified yet. With denosumab, interruptions in drug therapy during extensive surgery seem to reduce the risk of MRONJ, but the data are still deficient [6,16,45,46]. Otto et al. published an animal study in 2020 where 36 minipigs were divided into four different groups with regard to the success of a drug holiday under zolendronate therapy. Group 1 was the negative control (tooth extractions without zolendronate therapy), and group 2 was the positive control (tooth extractions under zolendronate without the recommended wound management). In group 3, tooth extractions were performed under the generally recommended conditions (smoothing of sharp bone edges, saliva-proof wound closure, antibiotic therapy) without AR interruption, whereas in group 4, the same surgical management was performed but with AR interruption 6 weeks preoperatively and 8 weeks postoperatively. In group 3, 83% of the pigs developed MRONJ, while in group 4, it occurred in 40% of the pigs [16]. This is an interesting finding that should be further investigated in other studies, including clinical studies, in order to generate more precise guidelines on the timing, duration and purpose of AR interruption. 

The preservation of the bone after tooth extraction, respectively the healing of the socket, is still of utmost importance. In addition to many other techniques, the use of autologous platelet concentrates (APCs) has been available for more than two decades. A systematic review included 43 studies to determine whether the use of APCs can be used effectively to prevent MRONJ. In 1219 extractions, APCs were used in 786 cases, and only 12 MRONJ cases were described. All patients had experienced high-dose administration. The results were not statistically significant, but this is an area where further controlled studies can investigate the potentially positive effect of APCs in preventing MRONJ [47]. In our study, no use of APCs was performed because of the actual low database of controlled studies.

However, we could not report significance in the development of MRONJ between malignant primary disease and osteoporosis nor a significant correlation between the manifestation of MRONJ and form of application (oral, subcutaneous, intravenous) or subcutaneous compared to intravenous application.

The risk varies between each patient, between malignant or benign groups and with the type of medication, but also depends on the drug itself [28,29]. Due to the binding of bisphosphonates to calcium ions, the associated intracellular increase leads to the interruption of the flow of cellular metabolites. This is caused by the closure of the so-called gap junctions (responsible for cell adhesion, among other things) and leads to epithelial collapse [2].

Since the expansion of the term into medication-related necrosis, the monoclonal antibody denosumab has played an important role. As known from the latest literature, the incidence of MRONJ among cancer patients receiving denosumab was 0.5–2.1% after one year, 1.1–3.0% after two years and 1.3% up to 3.2% after three years [30]. The half-life of denosumab, a human monoclonal antibody that binds to the receptor activator of nuclear factor-κB ligand (RANK-L) and affects osteoclast-guided bone resorption, is approximately 25–32 days [8,48]. Meta-analyses in the current literature describe a significantly higher risk of developing MRONJ under denosumab therapy than under zoledronate therapy [30]. Multiple myeloma (MM) patients have the highest risk of suffering from MRONJ (3.8–9.9%) [40] and intravenously applied zoledronate is most common and recommended in MM therapy [33,49]. In our patient population, however, we did not find a significant correlation between denosumab compared to zoledronate and MRONJ. Furthermore, although we found a significant difference between the underlying diseases and an MRONJ, especially when comparing breast carcinoma to renal cell carcinoma, this result should be interpreted cautiously due to the small group size and cannot be considered as evidence.

Since the pathological mechanisms for the development of MRONJ have not yet been fully clarified, it is assumed that the cause is multifactorial. Factors such as age, sex, diabetes or additional immunosuppressive therapy (e.g., steroids or antimetabolites, such as methotrexate (MTX) may play a role [6,8]. To date, it is only known that MTX or steroids that act as disease-modifying anti-rheumatic drugs (DMARDs) are possible indirect additional co-factors. Immunosuppression additionally favours infection, but invasive surgical interventions on the jaw must precede immunosuppressive therapy [34,43,50]. A study by Roman K. Rahimi-Nedjat et al. showed that diabetes could not be seen as an independent risk factor for the occurrence of MRONJ [51]. In our patient population, we were unable to show significant evidence of an association between MRONJ and diabetes or other co-factors but did show the significance of arterial hypertension being associated with MRONJ. However, this result needs to be substantiated by further studies and should be interpreted critically due to the retrospective nature of this study.

Another co-influencing factor might be nicotine abuse, which might favour the occurrence of MRONJ because of the additional decrease in oral TNF-α levels [52]. We were able to show significance in this regard in this study, but this observation should be viewed critically due to its retrospective character.

Regarding the treatment of MRONJ after it has occurred, a surgical approach is preferable to a conservative approach. In our population, no recurrences after MRONJ treatment occurred in seven of ten cases, whereas one received conservative therapy. However, preventive measures prior to the start of antiresorptive therapy were able to reduce the incidence of MRONJ after tooth extraction from 7.8% to 1.7% and are therefore recommended [53]. The interdisciplinary relationship between dentists and oncologists or orthopaedists should be promoted in every case. A detailed dental evaluation and diagnosis should be carried out in advance. A necessary tooth extraction should be performed by experienced surgeons or specialists under known special conditions, such as smoothening of the sharp bone edges, administration of perioperative antibiotics and primary wound closure without tension. In our study, all of the included patients did receive antibiotics (one patient denied it), and all of them underwent a standardised surgical procedure protocol.

There are shortcomings of this study that need to be discussed. First, due to the retrospective study design, there are discrepancies between the groups in terms of group size and composition, and therefore, statistical evidence is lacking. The interpretation of the observation of correlating co-factors, such as nicotine abuse and arterial hypertension, must be considered critical in this design of heterogenous groups. This also applies to the correlation of MRONJ and underlying diseases or gender, as the groups were only formed retrospective and not further subdivided. In particular, the significance levels from the statistical logistic regression should be viewed with caution overall because a larger sample size is required from the outset. Moreover, the extent and type of the surgical procedure varied between the groups, and since operations were performed by different surgeons, the surgical techniques varied to a certain extent. Furthermore, prospective studies are necessary to find further possible triggers or to verify other theories for the development of MRONJ.

## 5. Conclusions

Within this patient population, we were able to identify that the main risk factor for developing MRONJ was tooth extraction, especially in the lower jaw, without any correlation to the number of teeth or position (anterior vs. posterior) in the jaw. We were also able to show that the frequency of application of antiresorptive medication might be decisive for the development of MRONJ and should be kept in mind before extracting teeth. Even if there was no significant correlation between the different surgical procedures, the risk profile of developing MRONJ after dentoalveolar interventions can be expected as follows: tooth osteotomy > multiple extractions > single tooth extraction. However, further studies in terms of prospective study designs are needed to verify this profile.

## Figures and Tables

**Table 1:** Illustration of the patient cohort with *n* = 115 dentoalveolar interventions and relation to MRONJ+ occurrence with *n* = 10 from the total cohort.

	Groups	MRONJ (−) *n*	(%)	MRONJ (+) *n*	(%)
Total of interventions	115	105	91.30%	10	8.70%
age		68.69		61.80	
Gender	female (*n* = 90)	85	94.44%	5	5.56%
	male (*n* = 25)	20	80.00%	5	20.00%
Surgery	Single tooth extraction	53	94.64%	3	5.36%
	Multiple teeth extraction	34	89.47%	4	10.53%
	Osteotomy	18	85.71%	3	14.29%
Localisation	Maxilla	46	97.87%	1	2.13%
	Mandible	50	84.75%	9	15.25%
	Both (Mandible + Maxilla)	9	100.00%	0	0.00%
	Anterior	19	90.48%	2	9.52%
	Posterior	67	89.33%	8	10.67%
	Both	19	100.00%	0	0.00%
Underlying disease	Breast cancer	42	95.45%	2	4.55%
	Prostate cancer	12	92.31%	1	7.69%
	Osteoporosis	40	95.24%	2	4.76%
	Multiple myeloma	6	75.00%	2	25.00%
	Renal cell carcinoma	1	33.33%	2	66.67%
	Tonsils carcinoma	0	0.00%	1	100.00%
	Stromal tumour duodenum	1	100.00%	0	0.00%
	Malignant melanoma	2	100.00%	0	0.00%
	B-cell non-Hodgkin lymphoma	1	100.00%	0	0.00%
Other diseases	Heart diseases	9	100.00%	0	0.00%
	Arterial hypertension	35	83.33%	7	16.67%
	Diabetes	15	78.95%	4	21.05%
	Nicotine and alcohol abuse	2	50.00%	2	50.00%
	Thyroid disease	29	87.88%	4	12.12%
	Rheumatoid diseases	7	87.50%	1	12.50%
Antiresorptive medication	Zoledronate	51	91.07%	5	8.93%
	Alendronate	31	100.00%	0	0.00%
	Denosumab	7	70.00%	3	30.00%
	Ibandronate	9	100.00%	0	0.00%
	Pamidronate	5	71.43%	2	28.57%
	Risedronate	2	100.00%	0	0.00%
Form of application	Intravenous	65	90.28%	7	9.72%
	Oral	33	100.00%	0	0.00%
	Subcutaneous	7	100.00%	3	42.86%
Duration of medication	Mean (in months)	46.60		39.11	
	≤15 months	50	100.00%	0	0.00%
	>15 months	56	86.15%	9	13.85%
Frequency of application	Every 12 months	1	50.00%	1	50.00%
	every 6 months	12	100.00%	0	0.00%
	Every 3 months	12	85.71%	2	14.29%
	Every 4 to 6 weeks	36	85.71%	6	14.29%
	Every week	30	100.00%	0	0.00%

**Table 2:** Showing all statistical values among the patients with significance level *p* ≤ 0.05). Fisher’s exact test, chi-square test and Mann–Whitney U test were applied. After reaching a significance level, a logistic regression (marked in bold) was performed for a more precise determination.

Parameter	*p*-Value	Test
age	0.157	**
gender	0.038	*
**male vs. female**	**0.033**	****
Single tooth extraction	0.323	*
Multiple teeth extraction	0.728	*
Osteotomy	0.387	*
Preoperative antibiotic duration	0.497	**
Postoperative antibiotic duration	0.731	**
Total of extracted teeth	0.217	**
Localisation Maxilla/mandible	0.022	*
**Mandible vs. Maxilla**	**0.047**	****
Localisation Anterior/Posterior	0.849	***
Underlying disease treated with antiresorptive therapy	<0.001	***
**Renal cell carcinoma vs. breast carcinoma**	**0.009**	****
Arterial hypertension	0.035	*
Diabetes mellitus	0.059	*
Nicotine abuse	0.037	*
Antiresorptives	0.022	***
**Denosumab vs. Zoledronate**	**0.077**	****
Form of application	0.051	***
Subcutaneous vs. intravenous	0.098	***
Duration of application </> 15 month	0.608	*
Duration of application	0.859	**
Application frequency	0.038	***
4–6 weeks vs. 12 months	0.226	***

* Fisher’s exact test ** Mann–Whitney U test *** chi-square test **** blod: logistic regression.

**Table 3:** Illustration of MRONJ patients *n* = 10 and details (age, sex, underlying disease, medication, form of application, duration of medication, co-factors, localisation, surgery before MRONJ, surgery after MRONJ).

MRONJ Patient	1	2	3	4	5	6	7	8	9	10
Age	73	49	49	79	74	47	75	68	55	49
Gender	Male	Female	Female	Male	Female	Female	Female	Male	Male	male
Underlying disease	Prostate cancer	Breast cancer	Breast cancer	Multiple myeloma	Renal cell carcinoma	Secondary osteoporosis	Primary osteoporosis	Multiple myeloma	Renal cell carcinoma	Tonsils carcinoma
Medication	Zoledronate	Denosumab	Denosumab	Pamidronate	Zoledronate	Zoledronate	Pamidronate	Zoledronate	Denosumab	Zoledronate
Form of application	intravenous	subcutaneous	subcutaneous	Zoledronate	intravenous	intravenous	intravenous	intravenous	subcutaneous	intravenous
Duration of medication	70 months	24 months	30 months	72 months	16 months	48 months	40 months	n.a.	19 months	31 months
Co-Factors	Arterial hypertension	Arterial hypertension, Diabetes Thyroid disease	Arterial hypertension, Diabetes Thyroid disease	Diabetes, Renal disease	none	Arterial hypertension, Diabetes Thyroid disease	Arterial hypertension, Rheumatoid disease	Thyroid disease	Arterial hypertension	Arterial hypertension
Localisation	Mandible post.	Mandible post.	Mandible post.	Mandible post.	Mandible post.	Mandible post	Mandible post.	Mandible post.	Mandible post.	Mandible post.
Surgery before MRONJ	Single tooth extraction	Osteotomy	Multiple teeth extraction	Multiple teeth extraction	Osteotomy	Osteotomy	Single tooth extraction	Multiple teeth extraction	Single tooth extraction	Multiple teeth extraction
Surgery after MRONJ	1-fold sequestrectomy	1-fold sequestrectomy	1-fold sequestrectomy	conservative therapy (antibiotics, no surgery)	2-fold Sequestrectomy	1-fold sequestrectomy	3-fold Sequestrectomy	3-fold Sequestrectomy	1-fold sequestrectomy	1-fold Sequestrectomy

## Data Availability

The data presented in this study are available on request from the corresponding author.
